# Highly Sensitive Electrochemical Detection of Azithromycin with Graphene-Modified Electrode

**DOI:** 10.3390/s22166181

**Published:** 2022-08-18

**Authors:** Florina Pogăcean, Codruţa Varodi, Lidia Măgeruşan, Raluca-Ioana Stefan-van Staden, Stela Pruneanu

**Affiliations:** 1 National Institute for Research and Development of Isotopic and Molecular Technologies, 67-103, Donat Street, 400293 Cluj-Napoca, Romania; 2 Laboratory of Electrochemistry and PATLAB, National Institute of Research for Electrochemistry and Condensed Matter, 202 Splaiul Independentei Str., 060021 Bucharest, Romania; 3 Faculty of Applied Chemistry and Material Science, Politehnica University of Bucharest, 060042 Bucharest, Romania

**Keywords:** azithromycin, graphene-modified electrode, electrochemical detection

## Abstract

An electrochemical cell containing two graphite rods was filled with the appropriate electrolyte (0.2 M ammonia + 0.2 M ammonium sulphate) and connected to the exfoliation system to synthesize graphene (EGr). A bias of 7 V was applied between the anode and cathode for 3 h. After synthesis, the morphology and structure of the sample was characterized by SEM, XRD, and FTIR techniques. The material was deposited onto the surface of a glassy carbon (GC) electrode (EGr/GC) and employed for the electrochemical detection of azithromycin (AZT). The DPV signals recorded in pH 5 acetate containing 6 × 10^−5^ M AZT revealed significant differences between the GC and EGr/GC electrodes. For EGr/GC, the oxidation peak was higher and appeared at lower potential (+1.12 V) compared with that of bare GC (+1.35 V). The linear range for AZT obtained with the EGr/GC electrode was very wide, 10^−8^–10^−5^ M, the sensitivity was 0.68 A/M, and the detection limit was 3.03 × 10^−9^ M. It is important to mention that the sensitivity of EGr/GC was three times higher than that of bare GC (0.23 A/M), proving the advantages of using graphene-modified electrodes in the electrochemical detection of AZT.

## 1. Introduction

For many years, infectious diseases have represented a difficult, persistent, and on-going public health challenge. However, by the mid-1900s, following the introduction of antibiotics, the mortality rate of infectious illnesses had significantly decreased [1]. Selman Waksman used the word “antibiotic” for the first time to describe any tiny chemical generated by bacteria that inhibits the development of other microorganisms [2], and the “antibiotic golden age” (1940–1960s) saw the discovery of the majority of the antibiotic classes currently in use. They were hailed as a “medical miracle” because of their influence on the fate of humanity [3]. In order to kill germs or prevent them from proliferating, antibiotics—sometimes called antibacterials or antimicrobials—disrupt critical bacterial activities. Over time, bacteria develop resistance to antibiotics, which reduces their effectiveness [4]. Thus, in order to minimize antibiotic resistance, proper antibiotic usage is crucial.

Azithromycin (AZT), an azalide—a subclass of macrolide-type antibiotics, is a second-generation broad-spectrum antibiotic with a long half-life and a high tissue penetration degree [5], primarily administered in a wide variety of Gram-positive and Gram-negative bacterial infections, such as respiratory, dermal, genitourinary, sexually transmitted, and enteric infections. In addition to the antimicrobial properties, AZT has immunomodulatory and anti-inflammatory effects [6]. AZT has received increased attention because of additional benefits to stimulate defense reactions, being administered in chronic respiratory inflammatory diseases including diffuse panbronchiolitis, post-transplant bronchiolitis, and rosacea. Furthermore, the modulation of host responses facilitates its long-term therapeutic benefit in cystic fibrosis, non-cystic fibrosis bronchiectasis, exacerbations of chronic obstructive pulmonary disease (COPD), and noneosinophilic asthma [7]. Other recommended uses of AZT include the treatment of H. pylori infection; travelers’ diarrhea, and other gastrointestinal infections; Legionnaires’ disease (a type of lung infection); pertussis (whooping cough; a serious infection that can cause severe coughing); Lyme disease; severe infection or persistent lymphadenopathy due to Bartonella henselae (cat-scratch disease); and some tick-borne infections such as Australian tick typhus or scrub typhus. It is also employed as part of combined therapy for M. avium complex infections and babesiosis [8]. AZT is administered to prevent heart infection in people having dental or other procedures, and to prevent STD in victims of sexual assault. Nowadays, it is still unclear how AZT may affect both human and environmental health. Thus, in addition to more effective antibiotic usage management, there is also an urgent need to develop rigorous control methods to track medication concentrations in pharmaceutical formulations, biological fluids, or even in medium. The residues of antibiotics in the environment pose a potential health hazard. Among all antibiotics, AZT has one of the greatest concentrations in aqueous matrices [9]. Therefore, developing sensitive and selective analytical tools for AZY assay is essential. Until now, a variety of methods have been employed for this purpose, including: microbioassays [10,11]; high-performance liquid chromatography (HPLC) with ultraviolet detection or fluorescence detection [12,13,14]; gas chromatography with electron capture (GC–EC) or with mass spectroscopy (GC–MS) [15,16]; UV–Vis spectroscopy [17]; FTIR transmission spectroscopy [18]; spectrofluorometric method [19]; electrophoresis [20,21]; colorimetry [22]; and thin-layer chromatography (TLC) [23]. 

However, most of the reported methods present serious drawbacks since the microbiological approaches need long incubation periods and are affected by limited detection ability and poor accuracy [24,25], while the chromatographic and spectroscopic methods require complicated extraction or derivatization procedures that imply expensive and sophisticated equipment, high costs, and long-time analysis [26]. Under these circumstances, electrochemical assays have gained popularity due to the high sensitivity, ease of preparation, and real-time detection [27,28,29,30]. The electrochemical behavior of AZT has been related to the oxidation of tertiary amino groups [31]. Different voltammetric procedures have been applied for AZT electrochemical oxidation and quantification, including cyclic voltammetry and adsorptive stripping voltammetric determination [32,33]; linear sweep and differential pulse voltammetry [34]; and square wave striping voltammetry [35]. During the past decades, surface electrode modification represented a significant field in electrochemistry study and has been frequently employed to enhance electrochemical reactions [36,37]. A variety of surface modifiers have been used for AZT electrochemical assay: multiwall carbon nanotubes decorated with MgCr_2_O_4_ [38]; aniline [39]; renewable silver amalgam [40]; and ionic liquids [41]. Among the various types of surface modifiers, carbon-based materials, including graphenes, have brought major improvement in the development of electrochemical sensors [42,43].

With several surprising features in its two-dimensional structure [44], graphene has made its way into the mainstream of electrochemical analysis since it can enhance the modified electrodes’ performances by increasing the electron transfer efficiency, increasing the active area, as well as facilitating the interaction with the studied analyte [45]. However, its practical applicability is somewhat limited since simple methods for the mass production of high-quality large-area graphene have not been developed in an efficient manner. Since its discovery in 2004 [46], graphene production has followed several routes, namely: top-down (e.g., micromechanical cleavage [47]; electrochemical exfoliation [48]; exfoliation of graphite intercalation compounds [49]; solvent-based exfoliation [50,51]; exfoliation of graphite oxide [52]; arc discharge [53]; unzipping [54]) and bottom-up approaches (e.g., epitaxial growth [55]; chemical vapor deposition (CVD) [56,57]; flash pyrolysis [58]; reduction in carbon-containing species [59]). Despite so many existing methods, the large-scale production of high-quality graphene is still a challenge and, apparently, graphite electrochemical exfoliation offers the most suitable alternative for obtaining good quality materials in environmentally friendly conditions.

In this work, we present a simple approach for modifying a glassy carbon electrode with graphene obtained by a simple electrochemical method, without the use of organic solvents. The modified electrode exhibits improved characteristics both in terms of sensitivity and the detection limit of AZT, in comparison with bare GC. In addition, we used the standard addition method for the quantification of AZT in spiked samples, demonstrating excellent results.

## 2. Materials and Methods

### 2.1. Chemicals

High purity (99.995%) graphite rods (10 cm length; 6 mm diameter) were purchased from Sigma-Aldrich (Sternheim, Germany) and were employed during electrochemical exfoliation. Ammonium sulphate was bought from Reactivul Bucuresti (Bucharest, Romania), while the ammonia solution (25%) was purchased from POCH SA (Gliwice, Poland). Azithromycin (AZT) was purchased from HiMedia Laboratories Pvt. Limited (Mumbai, India), uric acid (≥99%, UA), dopamine hydrochloride (99%, DA), and dimethylformamide (DMF) were purchased from Alfa-Aesar (Karlsruhe, Germany), while L-tyrosine (Tyr) was purchased from Fluka BioChemika (Buchs, Switzerland). Glacial acetic acid (99.5%) was purchased from CHEMICAL COMPANY (Iași, Romania) and sodium acetate was purchased from Reactivul Bucuresti (Bucharest, Romania). Sodium dihydrogen phosphate (99.7%) and di-sodium hydrogen phosphate anhydrous (100%) were purchased from VWR Chemicals (Leuven, Belgium). Acetate buffer was employed for the preparation of the solutions with pH 3.6, 4.4, and 5. Phosphate buffer was employed for the preparation of the solutions with pH 6 and 7.

### 2.2. Standard Addition Method 

For azithromycin determination using the standard addition method, the following procedure was applied. In 5 mL of acetate buffer solution (pH 5), a proper amount (0.38 µL) from the commercially available pharmaceutical drug (SANDOZ; 100 mg/5 mL; 2.6 × 10^−2^ M AZT solution) was added, so the final concentration of the drug was 2 × 10^−7^ M AZT. This was denoted as the real concentration (C_real_) of the drug. Next, three equal volumes (50 µL) from 10^−4^ M AZT stock solution (in ethanol) were added to the beaker under continuous stirring and the current increase was read after each addition (+1.15 V applied potential). The current step was plotted versus the concentration, allowing us to determine the AZT (C_x_) in the analyzed drug, resulting in C_x_ = 1.76 × 10^−7^ M, while C_real_ = 2 × 10^−7^ M. The recovery was determined as the C_x_/C_real_ ratio (88.45%). It is important to mention that the recovery data obtained for several drug solutions of different concentrations ranged from 80 to 88.45%, indicating that some of the interfering species may interact with the electrode surface. According to the drug leaflet, the powder also contains sugar, xanthan gum, hydroxypropyl cellulose, anhydrous trisodium phosphate, anhydrous colloidal silicon dioxide, aspartame (E 951), caramel cream flavor, and titanium dioxide (E 171). 

### 2.3. Apparatus 

The morphological characteristics of the graphene sample were investigated with a Hitachi HD2700 instrument (Hitachi, Tokyo, Japan), equipped with a cold field emission gun (CSEG). 

X-ray powder diffraction (XRD) was employed for the structural investigations of the graphene sample. The sample was dispersed in DMF (0.2 mg/mL) and then deposited onto the surface of an amorphous quartz substrate. A D8 Advance Diffractometer (Brucker, Germany), 40 kV, 0.5 mA, equipped with a LYNXEXE detector (λ = 1.5406 Å) was employed.

The FTIR spectrum of graphene was recorded with a JASCO 6100 FTIR spectrometer (Jasco Inc., Easton, MD, USA), within a 4000–400 cm^−1^ spectral domain (4 cm^−1^ resolution). About 0.3 mg of graphene was mixed with KBr powder and pressed in a thin pellet, before recording the spectrum. 

A Christ-Alpha 1–4 LSC freeze dryer equipment (Martin Christ, Osterode am Harz, Germany) was employed for drying the graphene sample after electrochemical exfoliation of the graphite rods.

For the electrochemical measurements (differential pulse voltammetry (DPV) and amperometry), the cell containing three electrodes was coupled with a Potentiostat/Galvanostat Instrument PGSTAT-302N (Metrohm-Autolab B.V., Utrecht, The Netherlands). The GC (3 mm diameter) or the EGr/GC electrode was employed as a working electrode, together with a Ag/AgCl reference (3 M KCl) and a large foil platinum counter electrode. The DPV measurements were generally recorded from 0.5 to +1.5 V vs. Ag/AgCl, at a 10 mV/s scanning rate (0.05 s pulse time; 0.025 V pulse amplitude), while the amperometric measurements were recorded at +1.12 V (for the EGr/GC electrode) and +1.35 V (for the bare GC electrode). 

### 2.4. Synthesis of Exfoliated Graphene Sample (EGr)

An electrochemical cell containing two graphite rods (anode and cathode, 2 cm apart) was filled with the appropriate electrolyte (0.2 M ammonia + 0.2 M ammonium sulphate) and connected to the exfoliation system (a power supply, a static switch with adjustable parameters, and a Hewlett Packard 8005B pulse generator). The pulse generator allowed us to set the time parameters of the current pulses, such as the duration (0.8 s) and the pause between two pulses (0.2 s) [60]. During the exfoliation process, a bias of 7 V was applied between the anode and cathode (for 3 h). At the end, the material was washed with a large amount (8 L) of distilled water by decantation, filtered with Whatman qualitative filter paper (white ribbon), and finally dried by lyophilisation. Following this process, the sample was denoted EGr. 

### 2.5. Preparation of Graphene-Modified Electrode (EGr/GC)

N,N-dimethylformamide (DMF) was chosen as the organic solvent for the dispersion of the graphene sample (2 mg/mL). DMF has a higher boiling point (153 °C) in comparison with that of ethanol (78.37 °C) or distilled water (100 °C), which means that it evaporates slower at room temperature. Consequently, the graphene flakes from DMF solution have a longer time to interact by π–π stacking with the electrode surface, leading to the formation of a very stable layer on top of the GC electrode. A total of 10 µL from the graphene dispersion in DMF, previously homogenized with an ultrasonic device (SONICS Vibra-Cell), was deposited by the drop-casting method onto the GC electrode and dried at room temperature for 24 h. After that, the modified electrode (EGr/GC) was used for AZT electrochemical detection and quantification.

## 3. Results and Discussion

### 3.1. Morphological and Structural Investigation of EGr Sample

**Figure 1:**
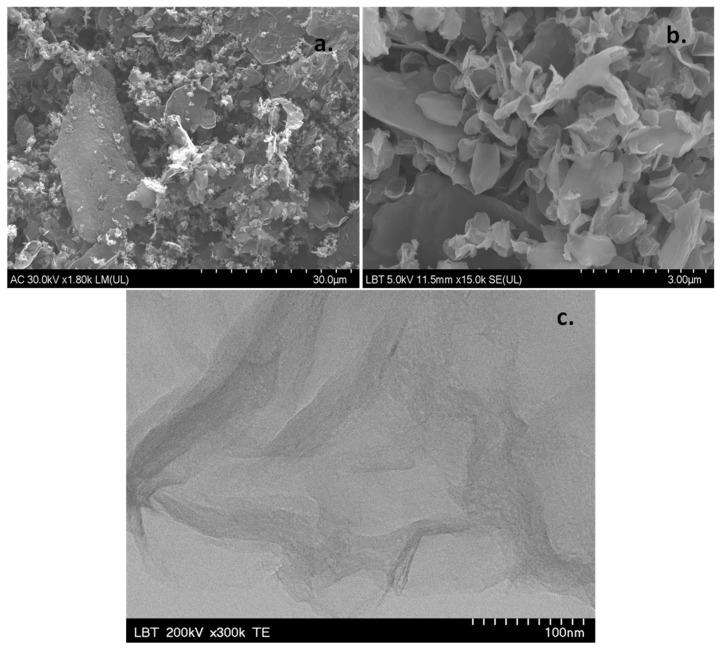
Representative SEM (**a**,**b**) and TEM (**c**) micrographs of the EGr sample; scale bar 30 µm (**a**); 3 µm (**b**); and 100 nm (**c**).

Figure 1 shows representative SEM/TEM micrographs of the graphene sample obtained after the exfoliation of the graphite rods. Here, one can see that some of the flakes are very large (e.g., 25 µm length; left side in Figure 1a) and have a smooth surface, while others are smaller and exhibit a wrinkled appearance. The edges of the flakes appear as bright lines. Figure 1b, obtained at higher magnification, reveals that the flakes are also thin and randomly oriented, generating a highly porous layer. The porous morphology of graphene deposited on top of a conductive substrate is highly beneficial in electrochemical investigations due to the increase in the active area of the modified electrode. The high transparency of graphene layers is clearly evidenced by the TEM micrograph presented in Figure 1c. 

**Figure 2:**
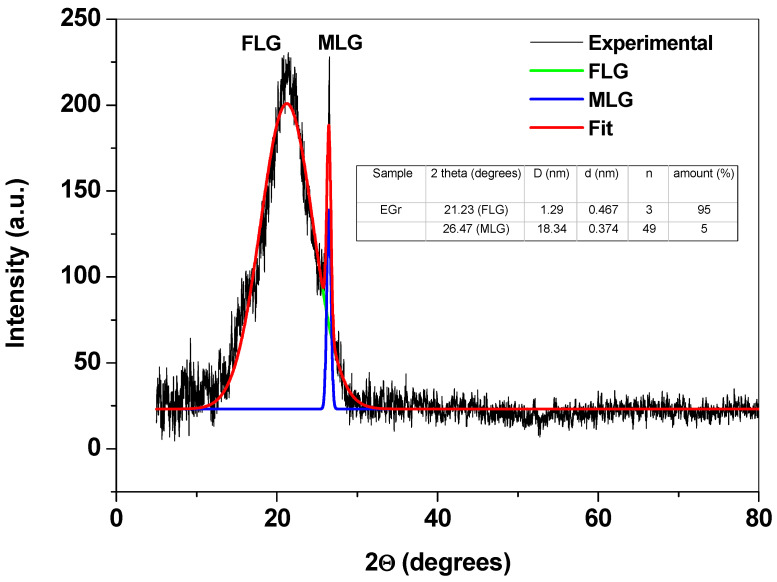
The XRD pattern of EGr sample dispersed in DMF and deposited onto a quartz substrate, showing the two peaks attributed to FLG and MLG; inset: the structural parameters determined from the XRD pattern: the mean size of graphene crystallite (D), the interlayer spacing (d), the average number of layers in graphene crystallites (n), and the amount (%) of FLG and MLG present within the sample.

The structural characteristics of the graphene sample were next investigated by the XRD technique. The pattern of the sample can be seen in Figure 2, evidencing two main peaks: the first one (at 21.23°) is very broad and corresponds to the reflections of few-layer graphene (FLG), while the second one (at 26.47°) is sharp and corresponds to the reflections of multilayer graphene (MLG). The amount of FLG present within the sample is predominant (95%) in comparison with MLG (5%) (see the inset of Figure 2). We also determined the mean size of graphene crystallite (D), the interlayer spacing (d), and the average number of layers present within the graphene crystallites (n) [61]. These values are also listed in the inset. It is interesting to observe that the interlayer spacing (d) is 0.467 nm in FLG and 0.374 nm in MLG, both being larger than the typical value in graphite (0.335 nm). The large interlayer spacing in FLG may be attributed to a higher number of oxygen-containing groups attached to the graphene sheets. FLG is the main product in the sample, and it is generated at the anode where the outer layers of graphite are electrochemically oxidized. In contrast, MLG flakes which have a smaller interlayer distance (less oxidized) are mainly generated at the cathode, due to gas evolution. 

**Figure 3:**
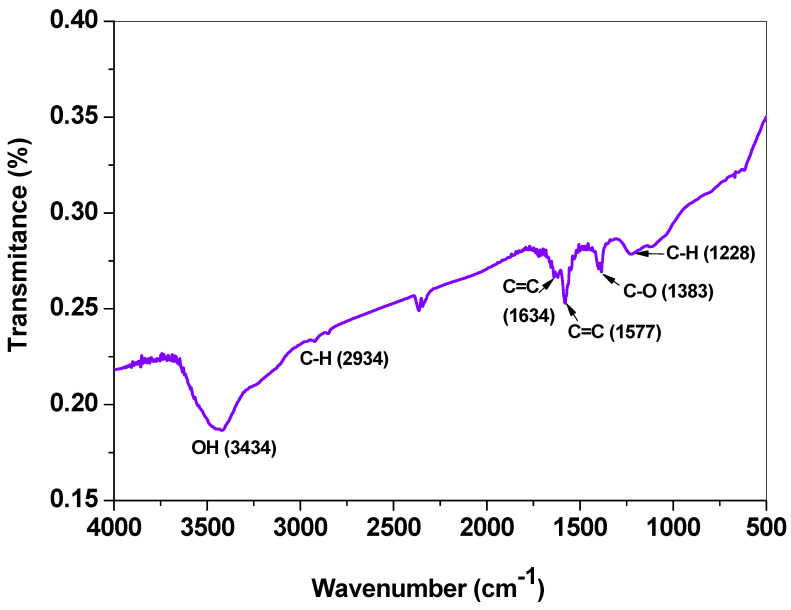
The FTIR spectrum of EGr sample embedded in KBr pellet.

Next, more information about the vibration characteristics of the functional groups attached to the graphene surface was obtained by the FTIR technique. The spectrum of the sample embedded in the KBr pellet is presented in Figure 3. In good agreement with the literature [62], the vibration bands of some oxygen-containing groups can be observed, but, due to the mild exfoliation process, their intensity is not very high. The broad band at around 3400 cm^−1^ can be assigned to the O–H stretching vibrations from the adsorbed water molecules, while the 1383 cm^−1^ band is assigned to the C–O stretching vibrations of the functional groups attached to graphene. The large shoulder at around 3000 cm^−1^ may be attributed to the aromatic C–H stretching bands, which are weak-to-moderate bands. The other two important bands are the aromatic ring stretching vibrations centred at around 1638 and 1577 cm^−1^, which generally appear as a pair of band structures. These bands are characteristic of the vibration modes of the sp^2^-hybridized carbon atoms (C=C). 

**Figure 4:**
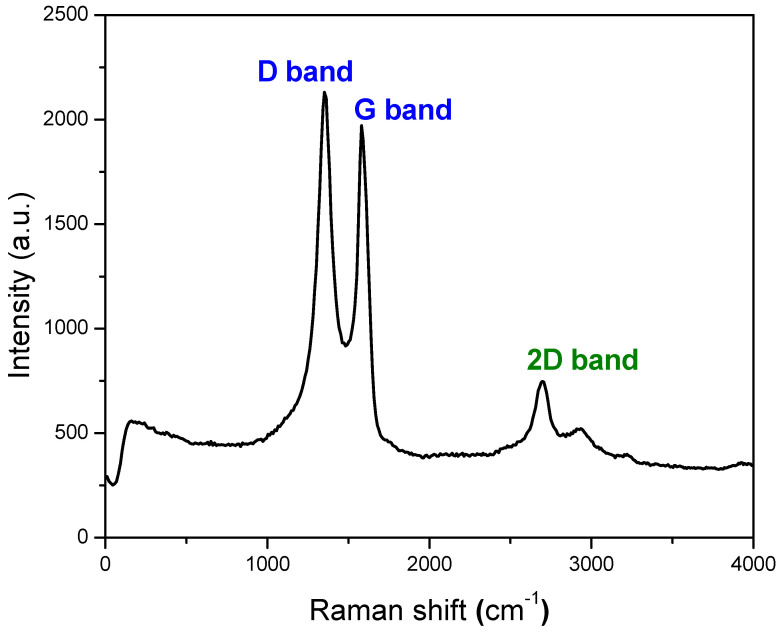
The Raman spectrum of EGr material.

Raman analysis is useful for determining the structural properties of graphitic materials. The defect (D) band at 1355 cm^−1^, the graphite (G) band at 1582 cm^−1^, and the 2D bands at 2708 cm^−1^, generated by the in-plane vibrations [63], shear modes [64], and the layer-breathing modes [65], are the most noticeable characteristics of the EGr spectrum (Figure 4). The Raman bands serve as a measure of the sample quality. In our case, the position of these spectral features is slightly shifted compared to other reported results [66], indicating the presence of some structural defects in the material [67]. However, the created defects can provide active sites for the electrochemical detection of AZT. The disorder extent was quantified based on the I_D_/I_G_ ratio [68], which was found to be 1.073. According to the Tuinstra and Koenig Equation (1) [69], the I_D_/I_G_ ratio is inversely correlated with the defect-free domain or the in-plane crystallite size (L_a_):

I_D_/I_G_ = C(λ)/L_a_ (1)

where C(λ) is a constant that depends on the radiation wavelength. L_a_ was determined to be 17.91 nm. This is a large value, so we can assume that the majority of the defects are located at graphene edges. This is in excellent agreement with the SEM/TEM micrographs, which revealed that the basal planes of graphenes are generally smooth and defect-free. 

### 3.2. Electrochemical Studies

**Figure 5:**
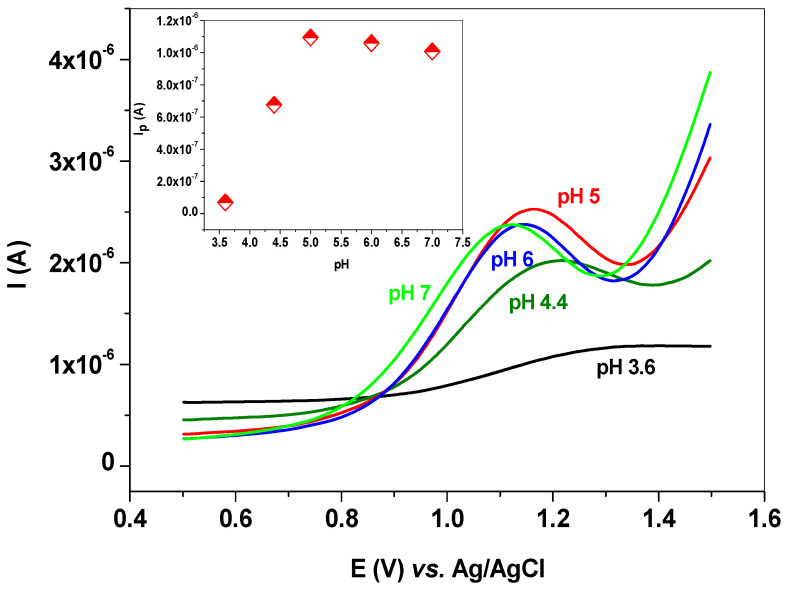
DPV signals recorded in supporting electrolyte of various pHs (3.6–7), each containing 10^−5^ M AZT; acetate buffer solutions (pH 3.6, 4.4, and 5) and phosphate buffer solutions (pH 6 and 7); inset: variation of peak current, I_p_, with the solution pH.

The first experiments with EGr/GC electrodes were focused on studying the effect of the solution pH on the electrochemical response of AZT (10^−5^ M). The DPV technique was employed, and the selected pH range was 3.6–7.0. Since the pKa of azithromycin is 7.34 [70], this molecule exists in the cationic form at pH < pK_a_. As can be seen in Figure 5, in highly acidic solution (pH 3.6), the peak potential is very high (+1.35 V) and shifts towards lower values (+1.1 V) in neutral solution. The peak current (I_p_) is well-defined above pH 4.4 and has a maximum value at pH 5. At pH value close to pK_a_, the peak current starts to decrease (see the inset of Figure 5) due to the slight deprotonation of AZT.

**Scheme 1:**
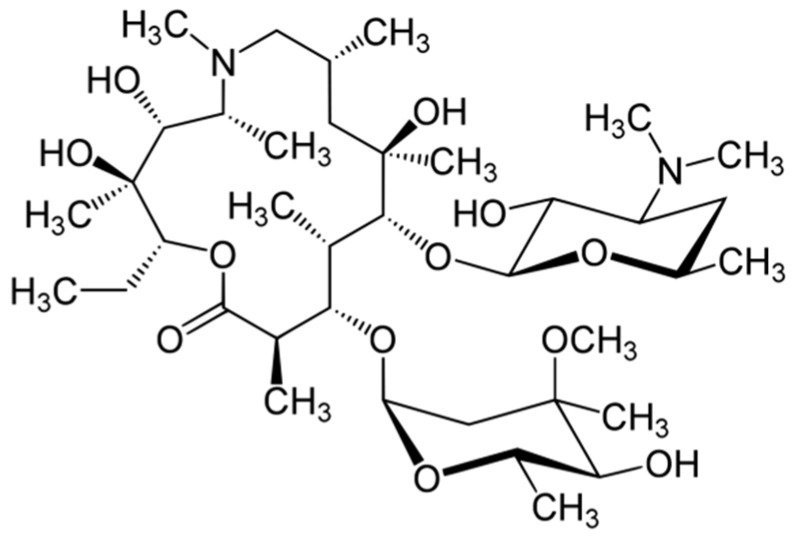
The chemical structure of azithromycin.

As can be seen in Scheme 1, azithromycin has various functional groups (hydroxyl, amine) that can be electrochemically oxidized. Generally, the amine group is easily oxidized by the loss of one electron, generating a radical cation [71]. In the case of AZT, one of the nitrogen atoms (N9) is situated in the macrocyclic lactone ring and so its lone pair of electrons cannot be easily removed, in comparison with the electrons in N3’s lone pair set. Wang et al. [72] have studied the electrochemical oxidation of another drug, erythromycin, which has a similar structure to AZT but without a nitrogen atom in the macrocyclic lactone ring. They reported that erythromycin exhibits similar voltammetric behaviour and the observed oxidation peak was attributed to the loss of one electron from N3’s lone pair. Therefore, in solutions with pH < pK_a_, the electrochemical oxidation of AZT proceeds via the initial deprotonation of the N3 atom followed by the subsequent loss of one electron and the formation of a radical cation. The reaction is irreversible, with no peak in the reverse scan. 

According to the study of Montenez [73], AZT is a hydrophobic molecule, due to the partial shielding of the N3 amino group by the methyl groups. Since graphene also has hydrophobic regions, especially the basal planes of the layers, AZT may interact by hydrophobic forces with the electrode surface and oxidation half reactions are facilitated. 

According to the literature [73], in solutions with pH < pK_a_, the electrochemical oxidation of AZT proceeds via the initial deprotonation of the N3 atom, followed by the subsequent loss of one electron and the formation of a radical cation. The reaction is irreversible, with no peak in the reverse scan (according to the cyclic voltammetric measurements; data not shown).

In order to determine the number of electrons involved in the oxidation reaction, the Laviron Equation (2) was employed [74]. The equation shows the variation of the peak potential, E_p,_ with the natural logarithm of the scan rate, lnυ, for irreversible reactions (linear sweep voltammetry).

(2) $$E_{p}=E^{0\prime }+\frac{RT}{\alpha_{a}nF}\ln\frac{RTk_{s}}{\alpha_{a}nF}-\frac{RT}{\alpha_{a}nF}\ln\upsilon$$

where α_a_ is the charge transfer coefficient, *k_s_*, the standard rate constant of the surface reaction, *n* is the number of electrons involved in the reaction, and *E*^0^′ is the formal potential.

**Figure 6:**
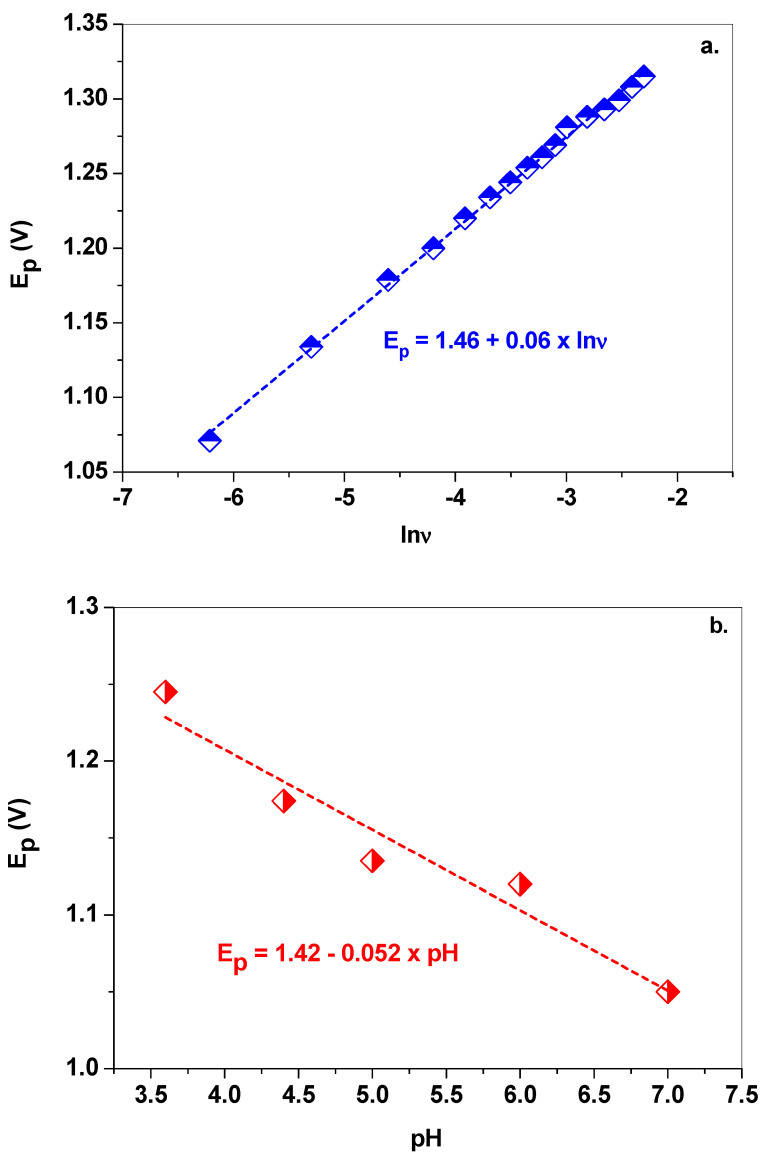
Dependence of peak potential, E_p_, versus the natural logarithm of scan rate, lnυ (**a**); dependence of E_p_ versus the pH (**b**).

LSVs were recorded in the acetate buffer (pH 5) solution containing 6 × 10^−5^ M azithromycin by varying the scan rate from 2 to 100 mV/s. Using the E_p_ vs. lnυ plot, we determined *n*α_a_ from the corresponding slope (Figure 6a). The obtained value was 0.42, being close to the 0.5 value of α_a_ in a totally irreversible electrode process. The above results demonstrate that one electron is involved in the electrochemical oxidation of azithromycin. 

The pH effect on the oxidation peak potential of azithromycin was also investigated (Figure 6b). The peak potential shifted negatively with a slope of −52 mV/pH indicating that protons take part in the oxidation process. A linear relationship between E_p_ and pH was obtained, following the equation: E_p_ = 1.42 − 0.052 × pH (r^2^ = 0.972). Since we determined that the number of electrons involved in the redox process is 1, it is reasonable to assume that the number of hydrogen ions taking part in the electrode reaction is also 1.

**Figure 7:**
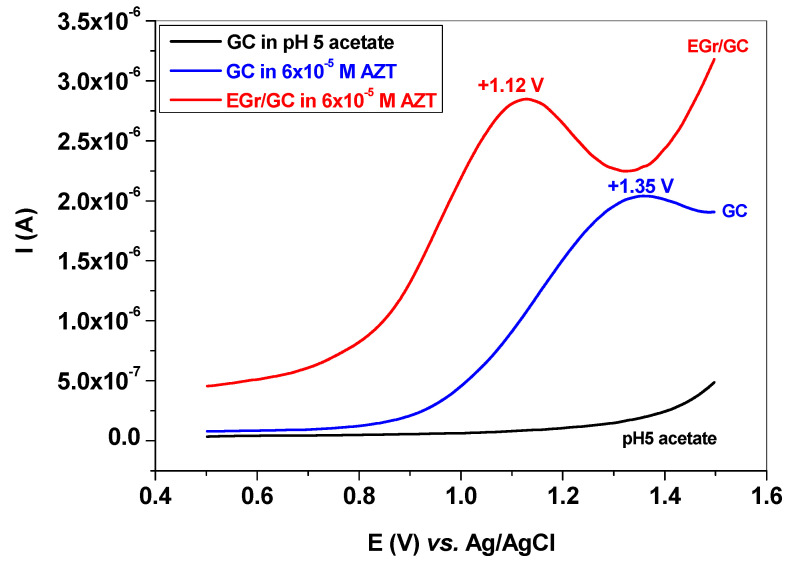
DPV signals recorded in pH 5 acetate containing 6 × 10^−5^ M AZT: EGr/GC (red curve) and GC (blue); GC in pH 5 acetate (black curve).

The electrocatalytic properties of the synthesized material towards AZT oxidation are now discussed in comparison with those of the bare GC electrode (Figure 7). Hence, the DPV signals recorded in the pH 5 acetate containing 6 × 10^−^^5^ M AZT revealed significant differences between the two electrodes. For EGr/GC, the oxidation peak is higher and appears at lower potential (+1.12 V) compared with that of the bare GC electrode (+1.35 V). The increased sensitivity of EGr/GC cannot be attributed only to its high electrochemical roughness factor, which was determined to be 1.46 (the ratio of the real—0.041 cm^2^ to the geometrical—0.028 cm^2^ area). Other factors are the enhanced electron transfer rate at the edges of graphene sheets and the hydrophobic interaction between AZT and graphene, which may help with the orientation of N3 amine group towards graphene, favouring its oxidation. 

**Figure 8:**
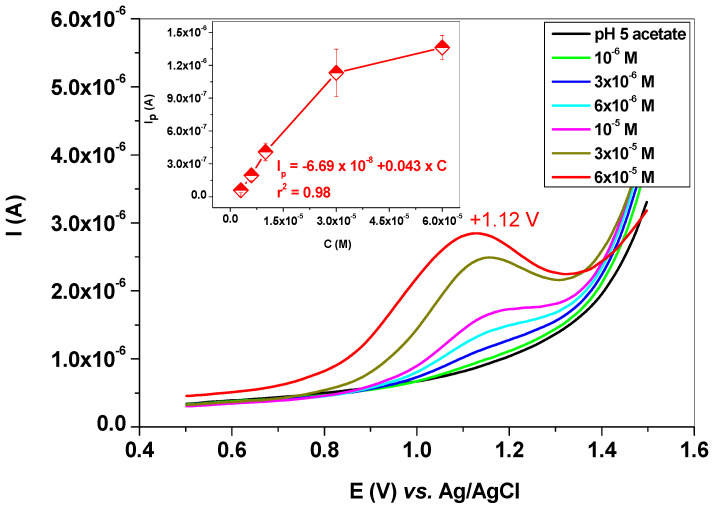
DPV recorded in supporting electrolyte of pH 5 containing various concentrations of AZT, from 10^−6^ to 6 × 10^−5^ M; inset: variation of peak current, I_p_, with the solution concentration, C.

Further analysis of AZT was investigated by recording the AZT signals at various concentrations (10^−6^–6 × 10^−5^ M) in pH 5 acetate buffer, using the DPV technique. The corresponding signals are plotted in Figure 8, while the inset shows the calibration plot obtained by representing the peak current vs. AZT concentration. It is interesting to note that the oxidation potential slightly changed with the concentration, indicating the adsorption of AZT/oxidation products on its surface. This is also reflected by the calibration plot (see the inset of Figure 8), where a saturation region can be observed above 3 × 10^−5^ M concentration. The linear range for AZT was 10^−6^–3 × 10^−5^ M, the sensitivity was 0.043 A/M, and the detection limit was 3.03 × 10^−7^ M. 

**Figure 9:**
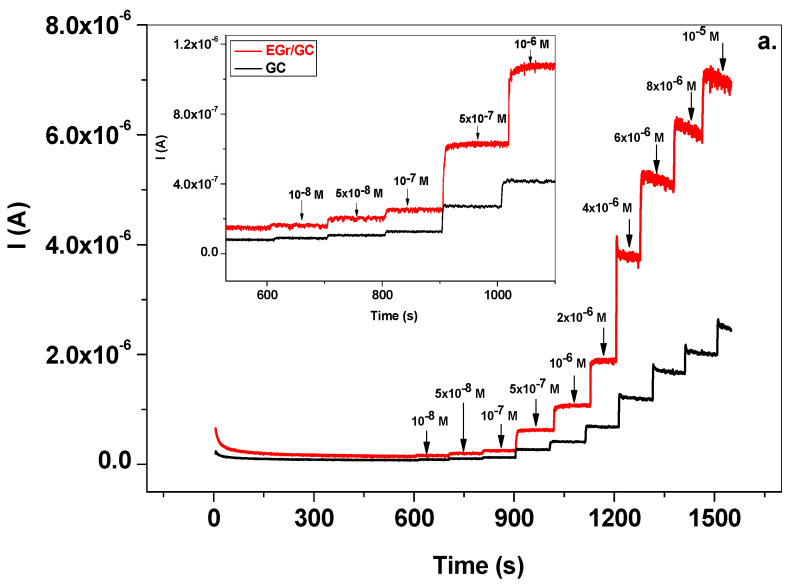

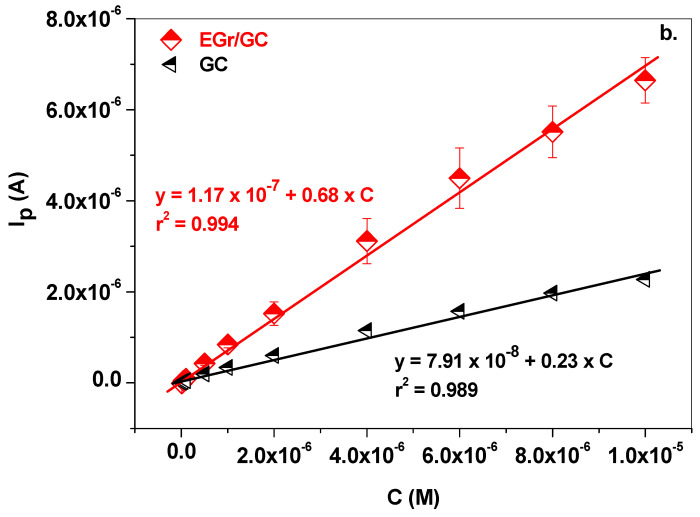
Amperometric curves recorded with EGr/GC (red) and GC (black) electrodes in supporting electrolyte of pH 5, after the addition of AZT from 10^−4^ M stock solution; the concentration range was from 10^−8^ to 10^−5^ M AZT; inset: the low concentration range, from 10^−8^ to 10^−6^ M (**a**); the corresponding calibration curves for EGr/GC (red) and GC (black) electrodes, using background subtracted signals (**b**).

Significantly better results were obtained by employing the amperometric technique (+1.12 V applied potential), as can be seen in Figure 9a,b. In this case, the linear range for AZT obtained with the EGr/GC electrode was very wide, 10^−8^–10^−5^ M, the sensitivity was 0.68 A/M, and the detection limit was 3.03 × 10^−9^ M. It is important to mention that the sensitivity of EGr/GC was three times higher than that of bare GC (0.23 A/M), proving the advantages of using graphene-modified electrodes in the electrochemical detection of AZT. 

**Figure 10:**
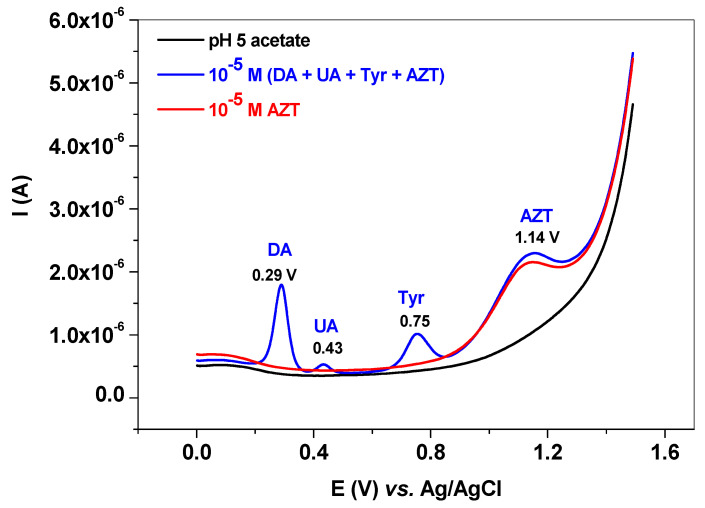
DPV signals recorded with EGr/GC electrode in pH5 acetate containing a single analyte (10^−5^ M AZT; red curve) and a mixture of analytes (DA + UA + Tyr + AZT; 10^−5^ M each; blue curve); 10 mV/s scanning rate.

Next, the selectivity of the EGr/GC electrode was evaluated in the presence of various interfering biomolecules such as dopamine (DA), uric acid (UA), and tyrosine (Tyr). In Figure 10, the DPV signals recorded for 10^−5^ M AZT (single analyte) and for a mixture of analytes, DA + UA + Tyr + AZT (10^−5^ M each), are presented. All the analytes appear at well-defined oxidation potentials and their mixture led to the slight increase in the AZT oxidation peak and potential, indicating a weak influence of the interfering species.

**Figure 11:**
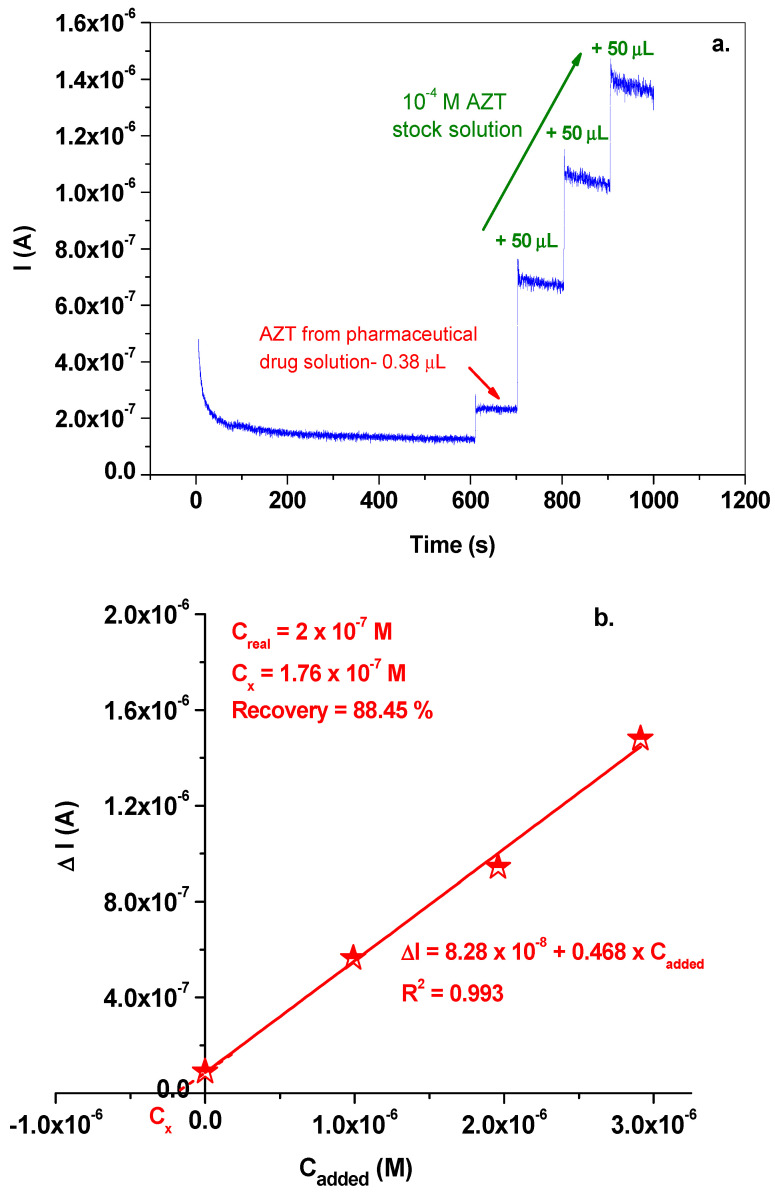
Amperometric curve recorded with EGr/GC electrode in pH 5 acetate after the addition of AZT pharmaceutical drug solution (0.38 µL; 2.6 × 10^−2^ M) and three additions of 50 µL of AZT from 10^−4^ M stock solution (**a**); the standard addition plot obtained for AZT quantitative analysis (**b**).

Since the EGr/GC electrode proved to have a good selectivity, it was additionally tested for AZT quantitative analysis in pharmaceutical drug solution, using the standard addition method (Figure 11a,b). The amperometric technique was employed since it was more sensitive compared with the DPV technique. The current step obtained after each AZT addition from the drug solution (2.6 × 10^−2^ M; 0.38 µL) and the stock solution (10^−4^ M AZT; three additions of 50 µL) was read and plotted versus the concentration, allowing us to determine the concentration of the drug, C_x_ (C_x_ = 1.76 × 10^−7^ M; C_real_ = 2 × 10^−7^ M; Recovery = C_x_/C_real_ = 88.45%). It is important to mention that the recovery data obtained for the three drug solutions ranged from 80 to 88.45%, indicating that some of the interfering species may interact with the electrode surface. 

Based on the experimental results obtained so far with EGr/GC, several advantages of the electrode can be mentioned: a simple approach was employed to prepare the graphene-modified electrode; the modified electrode has a very wide linear range (10^−8^–10^−5^) for AZT, with excellent sensitivity (0.68 A/M); and the detection limit was 3.03 × 10^−9^ M (see Table 1).

**Figure 12:**
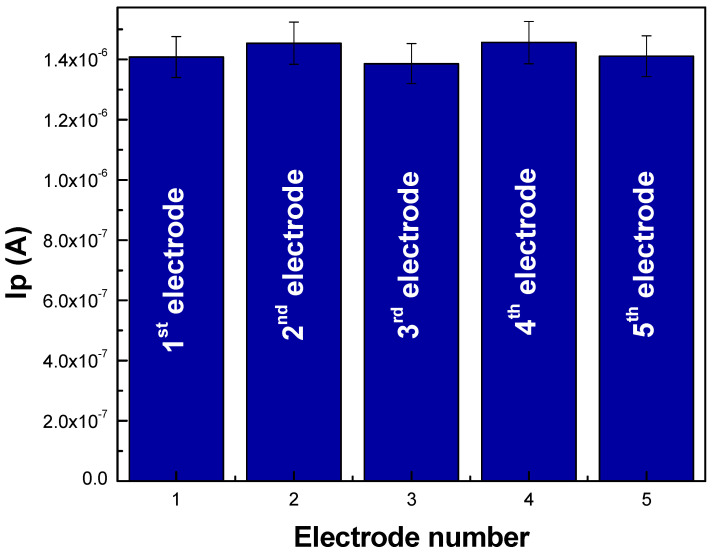
Variation of Ip recorded with five different modified electrodes in pH 5 acetate containing 6 × 10^−5^ M AZT.

The repeatability of the electrode modification method was evaluated by recording the DPV signal of five different electrodes in pH 5 acetate containing 6 × 10^−5^ M AZT (see Figure 12). The obtained results show a slight difference, smaller than 4.8%, in the recorded current intensity, proving that the method was reliable for electrode modification. 

**Figure 13:**
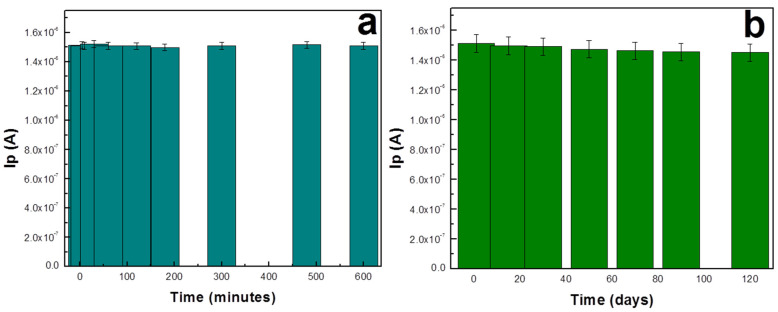
Intra- (**a**) and interday (**b**) DPV response of EGr/GC-modified electrode recorded in pH 5 acetate containing 6 × 10^−5^ M AZT.

In order to check the intra- and interday stability of EGr/GC and to prove the reproducibility of the AZT electrochemical detection method, the same modified electrode was subjected to repetitive DPV measurements, in replicate conditions, at different periods of time during a single day, covering a range of 10 h (see Figure 13a). As can be seen, the relative standard deviation between the different measurements was under 1.57%. Furthermore, the experiments were also reproduced on different days, over a long period of time, accounting for 120 days (see Figure 13b), with a very good reproducibility, since the differences in current intensity do not exceed 4.09%.

## 4. Conclusions

A graphene sample (EGr) was prepared by the electrochemical exfoliation of graphite rods and characterized by advanced techniques (SEM, XRD, and FTIR) to obtain information about its morphology and structure. The SEM micrographs revealed that some of the graphene flakes are large (e.g., 25 µm length) and have a smooth surface, while others are small (e.g., 2 µm length) and exhibit a wrinkled appearance. The flakes are also thin and randomly oriented, generating a highly porous layer which is beneficial in electrochemical investigations due to the increase in the active area of the modified electrode. The XRD technique evidenced that the sample contains few-layer graphene (95%) and multilayer graphene (5%), while the FTIR technique allowed the identification of the oxygen-containing groups attached to graphene. The graphene-modified electrode (EGr/GC) was next used for azithromycin (AZT) electrochemical detection and quantification. Excellent results were obtained by employing the amperometric technique (+1.12 V applied potential).

## Figures and Tables

**Table 1:** The comparison between the electrochemical performance of EGr/GC electrode with those of other modified electrodes employed for the determination of AZT.

Electrode/Sensing Material	Technique	Linear Range (M)	DL (M)	Sensitivity (A/M)	Ref.
VS2 QDs/cCNTs/N,	DPV	0.28–30 × 10^−8^	0.091 × 10^−8^	6.53	[75]
MIP/GNU/GO/GCE	DPV	0.3–920 × 10^−9^	0.1 × 10^−9^	0.0378	[39]
Gr/IL/GCE	DPV	6.54–38.1 × 10^−7^	2.53 × 10^−7^	-	[41]
Hg(Ag)FE	SWV SW-AdSV	6.42–31 × 10^−6^	1.92 × 10^−6^	-	[40]
		1.33–3.28 × 10^−6^	4 × 10^−7^	-	
CNFs-2/GCE	ECL	8 × 10^−8^–10^−4^	6.52 × 10^−8^	-	[76]
GO-MWCNTs/GCE	LCV	10^−7^–10^−5^	7 × 10^−8^	0.48	[42]
EGr/GC	Amperometry	10^−8^–10^−5^	3.03 × 10^−9^	0.68	This work

## Data Availability

Data will be provided upon reasonable request to the corresponding author.
